# A functional interaction between Hippo‐YAP signalling and SREBPs mediates hepatic steatosis in diabetic mice

**DOI:** 10.1111/jcmm.14262

**Published:** 2019-03-01

**Authors:** Zhiping Shu, Yuan Gao, Guopeng Zhang, Yu Zhou, Jing Cao, Dongyi Wan, Xiaohua Zhu, Wenqian Xiong

**Affiliations:** ^1^ Department of Nuclear Medicine Tongji Hospital, Tongji Medical College, Huazhong University of Science and Technology Wuhan China; ^2^ Obstetrics and Gynecology Union Hospital, Tongji Medical College, Huazhong University of Science and Technology Wuhan China

**Keywords:** hepatic steatosis, Hippo signalling, SREBPs, transcriptional activity, YAP

## Abstract

The Hippo pathway is an evolutionarily conserved regulator of organ size and tumorigenesis that negatively regulates cell growth and survival. Whether the Hippo pathway regulates cell metabolism is unknown. Here, we report that in the nucleus of hepatocytes, Yes‐associated protein(YAP)—the terminal effector of the Hippo pathway—directly interacts with sterol regulatory element binding proteins (SREBP‐1c and SREBP‐2) on the promoters of the fatty acid synthase (FAS) and 30‐hydroxylmethyl glutaryl coenzyme A reductase (HMGCR), thereby stimulating their transcription and promoting hepatocyte lipogenesis and cholesterol synthesis. In diet‐induced diabetic mice, either Lats1 overexpression or YAP knockdown protects against hepatic steatosis and hyperlipidaemia through suppression of the interaction between YAP and SREBP‐1c/SREBP‐2. These results suggest that YAP is a nuclear co‐factor of SREBPs and that the Hippo pathway negatively affects hepatocyte lipogenesis by inhibiting the function of YAP‐SREBP complexes.

## 1 INTRODUCTION

Obesity and type 2 diabetes are characterized by insulin resistance, non‐alcoholic fatty liver disease and dyslipidaemia, which leads to an increased risk of cardiovascular disease.^1^ Sterol regulatory element binding protein (SREBPs) are key lipogenic transcription factors that are mainly comprised of SREBP‐1c and SREBP‐2 in liver.^2^, ^3^ SREBP‐1c and SREBP‐2 regulate lipogenic process and cholesterol homeostasis respectively, by activating genes involved in these processes.^4^ When activated by insulin, the precursors of SREBPs migrate from the endoplasmic reticulum (ER) membrane to the Golgi and undergoes sequential proteolytic processing to release the transcriptionally active N‐terminal basichelix‐loop‐helix (bHLH‐Zip) domain. The active forms of SREBP‐1c and SREBP‐2 translocate into the nucleus, bind to sterol regulatory elements (SREs) present in the promoters of their own and target genes, and activate the transcription of SREBP‐responsive genes, thereby promoting the lipogenic process in the liver.^5^ The pathogenesis of hepatic steatosis, dyslipidaemia, and type 2 diabetes is closely related to the dysregulation of SREBPs.^6^

The Hippo pathway was initially defined by genetic studies in Drosophila melanogaster, which is an evolutionarily conserved controller of both cell proliferation and apoptosis.^7^ Sterile 20‐like kinases Mst1 and Mst2 (Hippo in *Drosophila*), large tumour suppressors Lats1 and Lats2 (Warts in *Drosophila*), Yes‐associated protein YAP (Yorkie from *Drosophila)* and its PDZ‐binding motif‐containing paralogue TAZ represent the core components of the mammalian Hippo pathway. Mst1/2 phosphorylate and activate Lats1/2 kinases, which form a complex with a regulatory protein Mob1 and further induce phosphorylation, nuclear exclusion and proteolytic degradation of YAP/TAZ. As the co‐activators of the Hippo pathway, YAP/TAZ acts mainly through binding to TEAD family transcription factors to promote proliferation and inhibit apoptosis.^8^, ^9^

YAP (Yorkie) and TAZ, relying on the regulation of Hippo pathway activity, control organ size and tissue homeostasis both in Drosophila and in mammals. Overexpression of YAP (Yorkie) or inactivation of the Hippo signalling pathway results in massive tissue overgrowth and leads to progression of tumorigenesis by promoting cell proliferation.^8^, ^10^, ^11^ Moreover, recent researches have shown that other physiological processes, including cell differentiation, stem cell self‐renewal, reprogramming and patterning, are also associated with the Hippo pathway.^7^, ^14^, ^15^ A key issue regarding to the Hippo signalling pathway is how this pathway cooperates with other signalling pathways in regulating a variety of processes.

Recent studies show that in transgenic mice, YAP promotes liver enlargement in a versible manner,^16^ suggesting that liver size control relies on tight regulation of Hippo pathway activity. Whether YAP affects the hepatocytes metabolism in this process needs further research. In addition, YAP/TAZ activation in tumour cells is promoted by increased levels of mevalonic acid produced by SREBPs transcriptional activity, which is induced by its oncogenic cofactor mutant p53.^17^ Furthermore, Lats2, a component of Hippo signalling, has been shown to suppress hepatic cholesterol accumulation by inhibiting SREBPs. As there are some connections between Hippo pathway and SREBPs, it is interesting for us to explore the role of Hippo‐YAP signalling in the lipid and cholesterol metabolism in liver.

Here, we show that SREBPs act as downstream effectors of Hippo‐YAP signalling to regulate the triglyceride and cholesterol metabolism of hepatocytes. Activation of Hippo signalling by ad‐Lats1 or sh‐YAP ameliorates insulin resistance, hepatic steatosis and hyperlipidaemia in diabetic mice.

## 2 METHOD

### 2.1 Animal protocols and diet

Male 8‐week‐old C57BL/6J background was purchased from BEIJING HUAFUKANG BIOSCIENCE COMPANY and kept on a 12 hours light cycle and given free access to food and water in a dedicated pathogen‐free animal facility at Huazhong University of Science and Technology (Wuhan, China). All animal experiments were approved by the Animal Care and Use Committee of Wuhan Union Hospital and were conducted in accordance with the National Institutes of Health Guidelines. All mice were divided into three groups: a normal chow diet containing 4% fat (wt/wt) and 72% carbohydrate, a high fat/high sucrose (HFHS) diet and a HFHS diet supplemented with the ad‐Lats1 or sh‐YAP. The viruses were administrated via caudal vein injection with 5 × 10^9^ pfu viruses of either control viruses or ad‐Lats1/sh‐YAP in a final volume of 200 µL of sterile NaCl 9% per mouse/month. The HFHS diet containing 35.5% fat (primarily lard), 36.6% carbohydrate (primarily sucrose) and no cholesterol (No. F1850, Bioserve, Frenchtown, NJ). Mice were maintained on the treatment for 16 weeks and afterwards were killed under isoflurane anaesthesia. Tissues were rapidly taken, freshly frozen in liquid nitrogen and stored at −80°C until needed for immunoblot analysis. Other parts of tissues were fixed for histological and immunohistochemical analysis.

### 2.2 Measurement of blood glucose, insulin level, plasma and liver lipids

Serum glucose concentrations were measured by FreeStyle blood glucose monitoring system (TheraSense, Alameda, CA). Plasma insulin levels were determined by ELISA (Linco Research, St.Charles, MO). The homeostasis model assessment of insulin resistance (HOMA‐IR) was calculated as previously described.^18^ Plasma, liver tissue and hepatocellular triglyceride and cholesterol levels were analysed as described.^19^

### 2.3 Primary mouse hepatocyte isolation and culture

Sodium pentobarbital (30 mg/kg intraperitoneally) were used to anaesthetize the C57BL/6 mouse. Primary mouse hepatocytes were isolated and cultured as described previously.^20^ Cell density was controlled at the same level by cell counting before the experiments.

### 2.4 Antibodies

Antibodies used for immunoblots were purchased from the indicated companies: p‐YAP (Ser127) (NO.13008), YAP (NO.14074), Lats1 (NO.3477), pLats1 (Ser909) (NO.9157), and pLats1 (Thr1079) (NO.8654) were from (Cell Signalling Technology). SREBP‐1 (Cat#557036), SREBP‐2 (Cat#557037) were from BD Biosciences.

### 2.5 Liver histological and immunohistochemical analysis

When experimental mice were sacrificed, livers of the mice were rapidly fixed in 10% phosphate‐buffered formalin acetate at 4°C overnight and embedded in paraffin wax. Paraffin sections (5 μm) were cut and mounted on glass slides. After dehydration, the haematoxylin and eosin and immunohistochemistry staining were made as according to the research.^19^

### 2.6 Plasmids and small interfering RNA

The mammalian expression vector for YAP (pcDNA3.0‐flag‐YAP1), SREBP‐1C (pcDNA3.1‐2 × flag‐SREBP‐1C), SREBP2 (pcDNA3.1‐2 × flag‐SREBP‐2) and Lats1 (pcDNA Lats1), mut‐YAP (pCMV‐flag S127A YAP) was from addgene.

siRNAsequence: siYAP: 5′‐GCACCUAUCACUCUCGAGA‐3′; siLATS1: 5′‐GAACCAAACUCUCAAACAA‐3′; si‐SREBP‐1c: 5′‐CAACCAAGACAGUGACUUC‐3′; si‐SREBP‐2: 5′‐CAACAGACGGUAAUGAUCAUU‐3′.

### 2.7 Adenovirus constructs

Adenovirus harbouring Lats1 (ad‐Lats1) and adenovirus harbouring shRNA for YAP (Ad‐sh‐YAP) were made according to previous research.^21^, ^22^Adeno‐X Maxi Purification Kit (Clontech) was used to purify the adenoviruses.

### 2.8 Chip and sequential chip

ChIP and sequential ChIP were conducted according to previous research.^22^

ChIP‐PCR primers: FAS‐PROMOTER: forward: CTCTCTGGCTCCCTCTAGGC, reverse: GATGGCCGCGGTTTAAATA; HMGCR‐PROMOTER: forward: TGCTGGGACTCGAACGGCTAT, reverse: TTACGCACGCTCGGAGCTGGAC；SREBP‐1C‐PROMOTER: forward: GCTCAGGGTGCCAGCGAACCAGTG, reverse: GGGTTACTAGCGGACGTCCGCC; SREBP‐2‐PROMOTER: forward: CAGGCATTCGCTCCGAGGC, reverse: TTGTTGTCAATGGGACCAG.

### 2.9 Immunoprecipitation

Primary mouse hepatocytes lysates were prepared using lysis buffer (50 mmol/L Tris (pH 7.4), 150 mmol/L NaCl, 1% Triton X‐100 and 1 mmol/L EDTA)and incubated with specific antibodies and protein A/G‐Sepharose beads at 4°C overnight. After immunoprecipitation, the immunocomplexes were washed with lysis buffer three times and eluted with 2 × SDS sample buffer. Finally, the precipitates were then analysed by immunoblots.

### 2.10 Transient transfection and dual‐luciferase assays

The reporter gene plasmids including human SREBP‐1c‐luc, SREBP‐2‐luc (including three canonical SREBP‐1c ‐response elements [SRE]) and the SRE mutant promoter of SREBP‐1c‐luc and SREBP‐2‐luc in the luciferase reporter vector pGL3‐Enhancer (Promega) were previously described.^23^, ^24^ The FAS‐Luc and HMGCR‐Luc reporter plasmid were previously reported.^25^, ^26^ Cells were plated on 12‐well plates and transfected with indicated luciferase vectors and Renilla luciferase plasmid pRL‐SV40 (Promega) as an internal control, the ratio of luciferase vector and Renilla plasmid was 50:1. The transcriptional activity was determined using a luciferase assay system (Promega).

### 2.11 RNA isolation and quantitative RT‐PCR

Total RNA were isolated from cultured HepG2 cells or from mouse livers. Synthesized cDNA was subjected to real‐time PCR analysis.

Quantitative PCR primers:

Mouse: SREBP1a: forward: GGCCGAGATGTGCGAACT, reverse: TTGTTGATGAGCTGGAGCATGT;

SREBP1c: forward: GGAGCCATGGATTGCACATT, reverse: GGCCCGGGAAGTCACTGT; SREBP2: forward: GCGTTCTGGAGACCATGGA, reverse: ACAAAGTTGCTCTGAAAACAAATCA;

HMGCR: forward: CTTGTGGAATGCCTTGTGATTG, reverse: AGCCGAAGCAGCACATGAT; HMGCS: forward: GCCGTGAACTGGGTCGAA, reverse: GCATATATAGCAATGTCTCCTGCAA;

ACC1 forward: TGACAGACTGATCGCAGAGAAAG, reverse: TGGAGAGCCCCACACACA;

FAS: forward: GCTGCGGAAACTTCAGGAAAT, reverse: AGAGACGTGTCACTCCTGGACTT;

SCD1: forward: TTCTTCTCTCACGTGGGTTG, reverse: CGGGCTTGTAGTACCTCCTC.

Beta‐actin: forward: CCACAGCTGAGAGGGAAATC, reverse: AAGGAAGGCTGGAAAAGAGC.

Human: HMGCR: forward: TGATTGACCTTTCCAGAGCAAG, reverse: CTAAAATTGCCATTCCACGAGC;

FAS: forward: AGTACACACCCAAGGCCAAG, reverse: GGATACTTTCCCGTCGCATA.

Beta‐actin: forward: GATGAGATTGGCATGGCTTT, reverse: GTCACCTTCACCGTTCCAGT.

Values were standardized using β‐actin. Data were analysed using the ΔΔCT threshold cycle method.

### 2.12 Immunoblot analysis

Extracts of liver homogenates or cell lysates were prepared using the extraction reagent kit (Pierce Biotechnology Inc) according to the manufacturer's instructions. Equal amounts of protein (10‐20 µg) were subjected to 10%–15% SDS–PAGE, transferred to a PVDF membrane and probed with the indicated antibodies.

### 2.13 Statistical analysis

All values are expressed as mean ± SEM Statistical analysis between groups was conducted by unpaired Student's *t* test or one‐way analysis of variance (ANOVA) followed by a Newman‐Keuls comparison test. Values of *P* < 0.05 were considered to be significant.

## 3 RESULTS

### 3.1 YAP is dephosphorylated and translocates into nucleus of hepatocytes in HFHS‐fed mice

**Figure 1:**
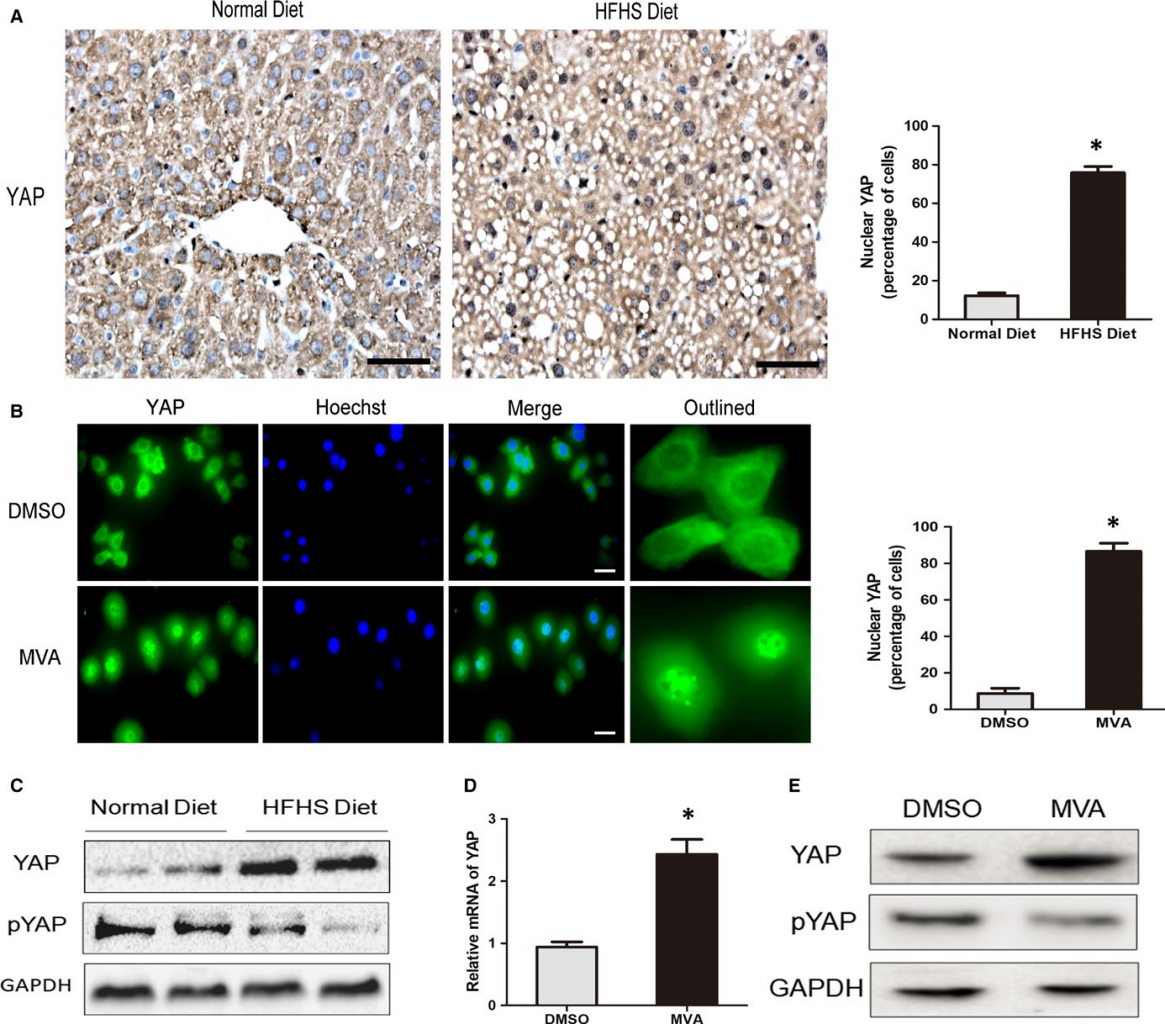
YAP protein is dephosphorylated and activated in liver of HFHS diet‐fed mice. A, Representative immunohistochemical staining for YAP protein in mice with normal diet or HFHS diet. YAP was accumulated in the nucleus of hepatocytes in mice with HFHS diet. Photographs were taken at magnifications of ×400. Nuclear YAP were quantified (mean ± SEM, *P < 0.05, vs Normal Diet, Scale bars, 50 μm). B, Primary hepatocytes from mice (n = 4) were treated with dimethylsulphoxide (DMSO) or with 0.5 mmol/L MVA for 24 h before fixation. Representative images of immunofluorescence for YAP protein were shown. Primary hepatocytes with nuclear YAP were quantified (mean ± SEM, *P < 0.05, vs DMSO, scale bars, 15 μm). C, Western blot analysis of YAP and pYAP (S127) in mice with normal diet or HFHS diet. D, The mRNA levels of YAP in primary hepatocytes from mice (n = 4) were detected by RT‐PCR (mean ± SEM, *P < 0.05, vs DMSO). E, Total YAP and pYAP (S127) protein were detected by Western blot

**Figure 2:**
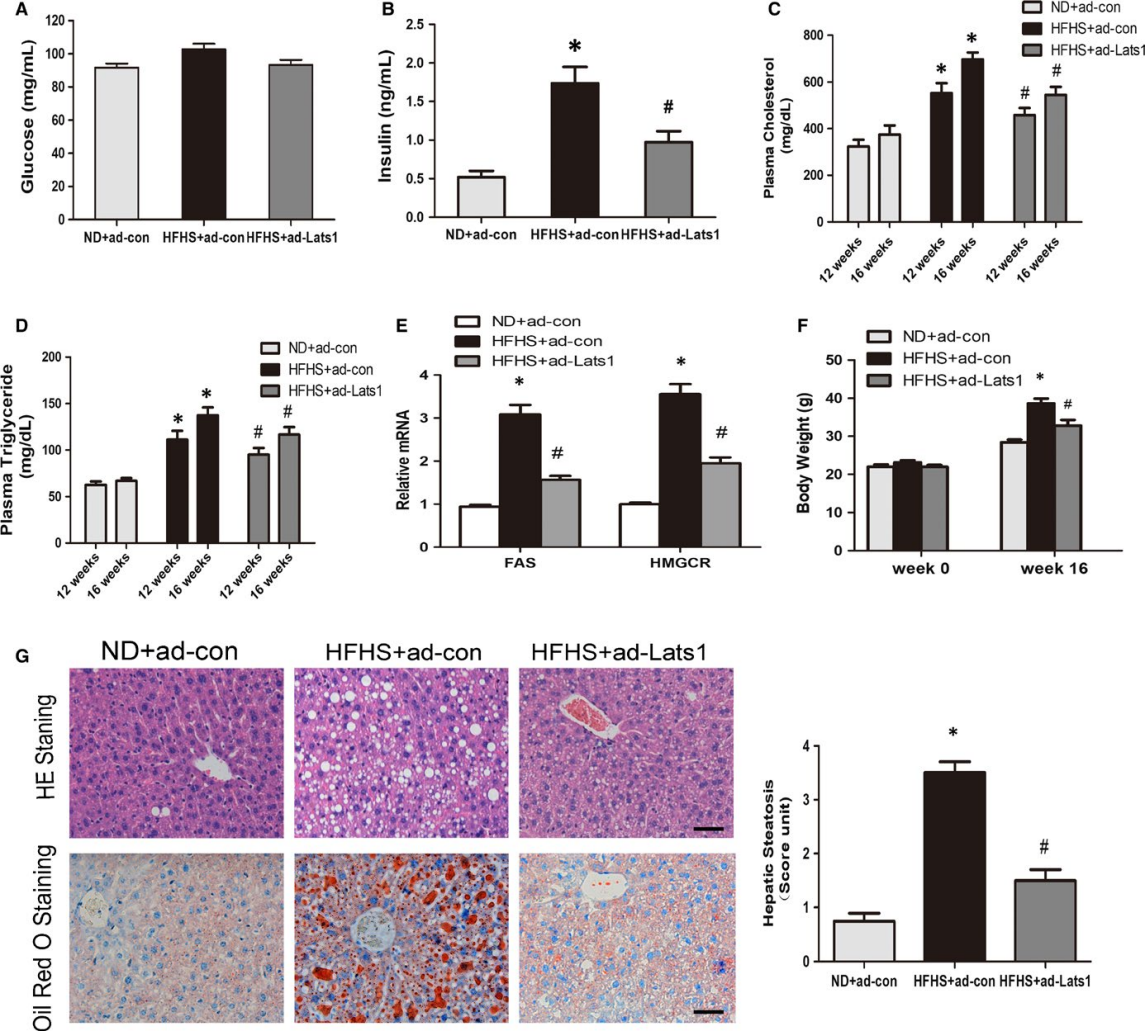
Lats‐1 protects against insulin resistance and hepatic steatosis in HFHS diet‐fed mice. A and B, Plasma insulin levels and blood glucose were assessed in mice fed a normal chow diet with ad‐con (n = 15), a HFHS diet with ad‐con (n = 30) and a HFHS diet with ad‐Lats1 (n = 30). C and D, Time course changes of plasma triglyceride and total cholesterol levels in mice following a 16 h fast (n = 8‐16). E and F, mRNA levels of FAS and HMGCR and changes of body weight in mice (n = 15‐30). G, Representative gross morphology of the mouse livers, Haematoxylin and eosin staining and oil red O staining of liver sections (n = 8‐16). Photographs were taken at magnifications of ×200. The degree of lipid infiltration on Haematoxylin and eosin staining was scored, scale bars, 50 μm. Data are shown as mean ± SEM, *P < 0.05, vs normal diet mice with ad‐con; ^#^ *P* < 0.05, vs HFHS‐fed mice with ad‐con

The diabetogenic diet composed of high fat and high sucrose (HFHS), previously described to promote obesity and type 2 diabetes in C57BL/6 mice, could induce hepatic steatosis, hyperlipidaemia, insulin resistance and enhanced atherosclerotic plaques in mice.^27^, ^28^ In order to explore the phosphorylation status of YAP in diabetic mice, C57BL/6 mice were placed on either a normal chow diet or a HFHS diet for 16 weeks. Immunohistochemistry of the liver showed YAP protein was mainly located in cytoplasm of hepatocytes in mice with normal diet. But in mice fed with HFHS diet, YAP protein accumulated and translocated into nucleus of hepatocytes (Figure 1A). Western blot was also performed and reflected that, in diabetic mice, the YAP protein was increased and dephosphorylated at serine 127 (Figure 2B), which promotes YAP nuclear translocation and activation.^7^ Furthermore, we cultured the primary hepatocytes of mice and stimulated the cells with mevalonic acid (MVA), which was accumulated in diabetic mice and reported to induced YAP nuclear localization and activation in tumour cells. Immunofluorescence showed YAP was mainly located in cytoplasm of primary hepatocytes and addition of MVA to the culture medium could induce nuclear YAP localization (Figure 1B). Also MVA stimulation dephosphorylated the YAP and increased its mRNA and protein levels apparently (Figure 1D,E). Taken together, these results suggested that YAP was dephosphorylated and translocated into nucleus of hepatocytes of diabetic mice and MVA may be one of the stimulators.

### 3.2 Phosphorylation of YAP by ad‐Lats1 improves systemic insulin resistance and hepatic steatosis in diabetic mice

Lats1, acting directly upstream of the Hippo pathway effector YAP, phosphorylates YAP at Ser127 and induces YAP nuclear exclusion and cytoplasmic retention.^31^ To research the role of Hippo pathway in the lipid metabolism, we first phosphorylated or knocked down YAP in mice fed with normal chow diet with adenoviruses harbouring Lats1 (ad‐Lats1) or adenoviruses harbouring shRNA (sh‐YAP). Results showed neither the plasma triglyceride and cholesterol levels nor the mRNA levels of FAS and HMGCR were not significantly changed (Supplementary Figure S1A,B). These phenomena may be due to the YAP protein mainly phosphorylated and accumulated in the cytoplasm, which was an inactivated state.

Then, we wanted to explore the role of Hippo pathway in the diabetic mice. C57BL/6 mice were fed with either a normal chow diet, a HFHS diet or a HFHS diet for 16 weeks and administrated with ad‐Lats1 or the control adenoviruses. As shown in Figure 2A,B, ad‐Lats1 caused an obvious reduction in plasma insulin and a mild decrease in plasma glucose in mice fed with HFHS diet and total plasma cholesterol and triglyceride levels at 12 weeks and 16 weeks apparently decreased by approximately 25% in diabetic mice after ad‐Lats1 administration (Figure 2C,D). The mRNA levels of FAS and HMGCR in liver, key enzymes for the synthesis of triglyceride and cholesterol, were also reduced by Lats1 overexpression (Figure 2E).Consistently, haematoxylin and eosin (H&E) staining and oil red O staining experiments indicated administration of ad‐Lats1 reduced excess fat accumulation in hepatic intracellular vacuoles (Figure 2G). Due to these physiology changes, the body weight of mice with HFHS diet was mildly decreased by the Lats1 overexpression (Figure 2F). These results demonstrated that ad‐Lats1 effectively improves HFHS diet‐induced change of plasma cholesterol and triglyceride levels, hepatic steatosis and insulin resistance in mice.

**Figure 3:**
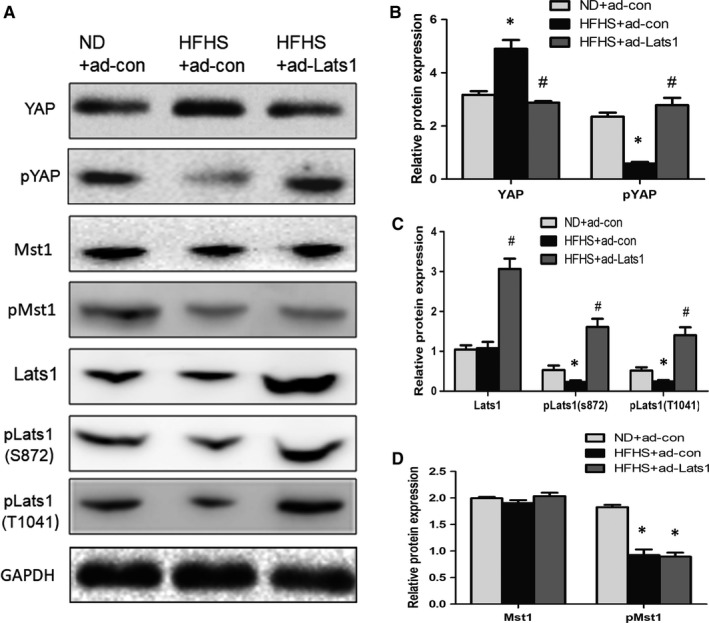
Hippo signalling is inactivated in the liver of diabetic mice. A‐D, Immunoblot analysis for YAP, pYAP (S127), p‐Lats1 (T1041 and S872), Lats1, p‐Mst1 (Thr183), Mst1 were detected. Quantification of the immunoblots were shown (*P < 0.05, vs normal diet mice with ad‐con; ^#^ *P* < 0.05, vs HFHS‐fed mice with ad‐con, mean ± SEM, n = 4)

Western blot showed HFHS diet induced inactivation and dephosphorylation of Mst1 and Lats1, increased total YAP protein and decreased pYAP protein, suggesting that Hippo signalling was inactivated in the liver of diabetic mice (Figure 3A‐D). Furthermore, ad‐Lats1 administration increased phosphorylated Lats1 and YAP protein in mice with HFHS diet, leading to Hippo signalling inactivation (Figure 3A,B). Taken into account previous results, these findings suggests that Lats1 and YAP phosphorylation in hepatocytes may contribute to the alleviated physiology alterations in diabetic mice.

### 3.3 Knockdown of YAP ameliorates aberrant lipogenesis and suppresses expression of SREBPs and their target genes in the livers of diabetic mice

**Figure 4:**
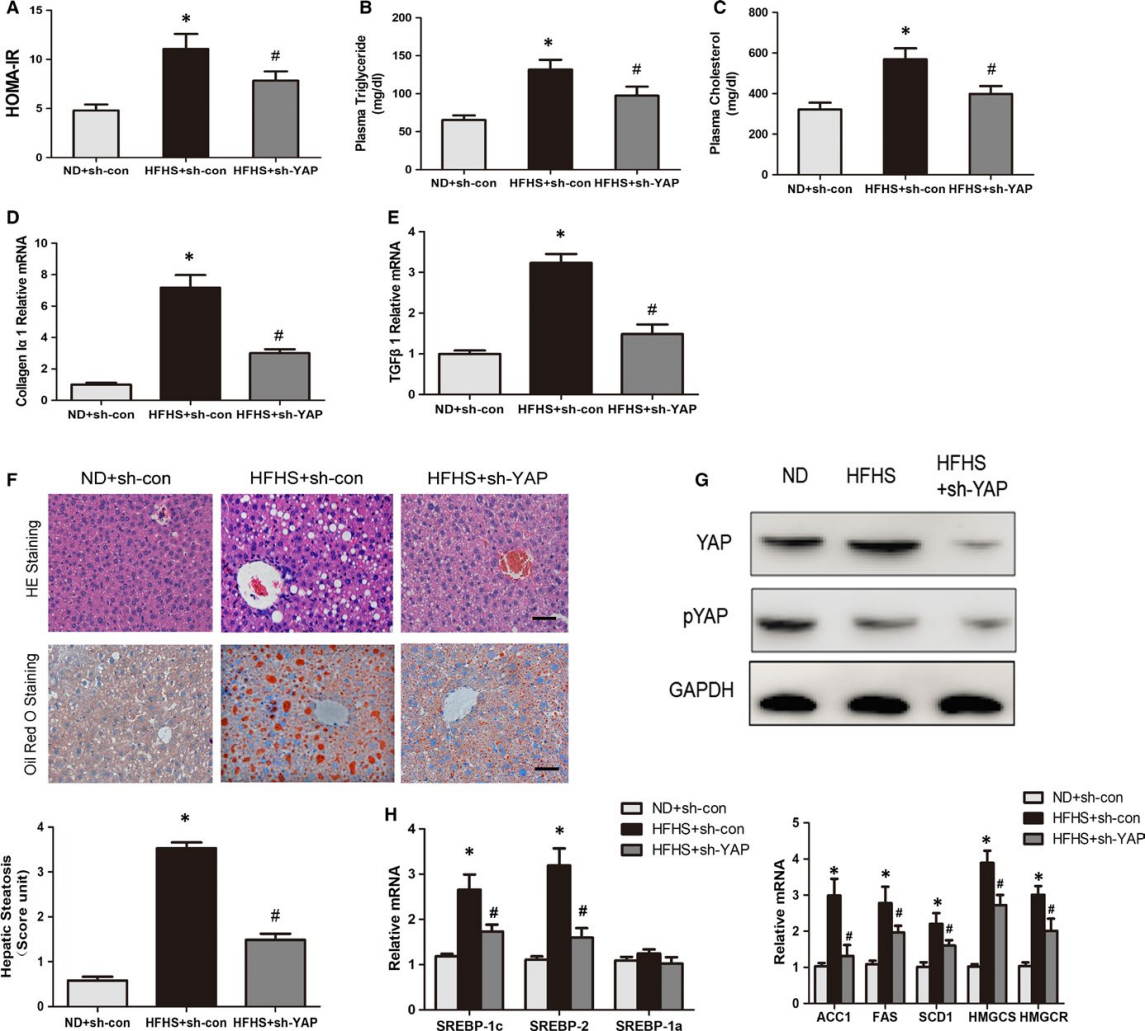
YAP knockdown by sh‐YAP inhibits expression of SREBPs and their target lipogenic enzymes, and reduces lipid accumulation in the liver of diabetic mice. A, Calculated HOMA‐IR were assessed in mice fed a normal chow diet with sh‐con (n = 15), a HFHS diet with sh‐con (n = 15) and a HFHS diet with sh‐YAP (n = 20). B and C, Plasma triglyceride and total cholesterol levels in mice following a 16 h fast (n = 8‐16). D and E, Gene expression changes related to fibrosis. Liver TGFβ1, and collagen Iα1 gene expression was determined by real‐time RT‐PCR (n = 10). F, sh‐YAP treatment inhibits lipid accumulation in the liver of HFHS‐fed mice. Representative gross morphology of the mouse livers, Haematoxylin and eosin staining and oil red O staining of liver sections. Photographs were taken at magnifications of ×200 (n = 8‐16). The degree of lipid infiltration on Haematoxylin and eosin staining was scored, scale bars, 50 μm. G, Immunoblot analysis for YAP and pYAP (S127) were detected. H, The transcription of genes involved in triglyceride and cholesterol biosynthesis is decreased in the liver of sh‐YAP–treated mice. The mRNA amounts of genes encoding SREBP‐1a, SREBP‐1c and SREBP‐2, as well as genes encoding ACC1, FAS, SCD1, HMGCS and HMGCR in the mouse livers, were determined by real‐time RT‐PCR (n = 8‐16). Data are shown as mean ± SEM, *P < 0.05, vs normal diet mice with sh‐con; ^#^ *P* < 0.05, vs HFHS‐fed mice with sh‐con

To determine whether the phenotypic alterations of ad‐Lats1–treated mice might be due to the phosphorylation of activated YAP in hepatocytes, we knocked down the YAP protein in mice through administration of sh‐YAP. C57BL/6 mice were fed with either a normal chow diet, a HFHS diet, or a HFHS diet for 16 weeks, and administrated with sh‐YAP or the control adenoviruses. Similar to previous results, sh‐YAP also caused a moderate decrease in the insulin resistance, as the calculated HOMA‐IR was lower in the sh‐YAP treated mice (Figure 4A). Administration of sh‐YAP also lowered the total plasma cholesterol and triglyceride levels in insulin resistant mice after 16 weeks of HFHS diet (Figure 4B,C). The expression of fibrogenic genes, collagen Iα1 and TGF‐β1, were also decreased by YAP knockdown in HFHS‐fed mice (Figure 4D,E). Haematoxylin and eosin staining and oil red O staining reflected the effective reduction of excess fat accumulation in hepatic intracellular vacuoles in sh‐YAP treated group (Figure 4F). Western blot revealed that the total YAP and phosphorylated YAP were apparently decreased, which contributed to the above changes (Figure 4G). Thus, these results demonstrated that YAP may function as the terminal effector of the Hippo signalling pathway in mediating the lipogenesis, cholesterol synthesis and hepatic steatosis in diabetic mice.

As important metabolic transcription factors, SREBPs play critical roles in the lipogenesis and cholesterol synthesis through activating the relevant enzymes in the insulin resistance mice.^32^ We have been suggested that the phenotypic alterations of ad‐Lats1 or sh‐YAP–treated mice might be owed to the inhibition of hyperactive SREBPs in hepatocytes. Previous researches show that the mRNA and protein levels for SREBPs could be increased by themselves via a feed‐forward mechanism.^2^ As the antibody used for the Western blot could recognize both SREBP‐1c and SREBP‐1a isoforms, we determined the expression of SREBPs with real‐time PCR. Furthermore, the mRNA levels of SREBP‐1c and SREBP‐2, mainly expressed in the liver, and SREBP‐1a, the less abundant isoform, were detected by real‐time PCR. As SREBP‐1a mRNA was not affected by either hyperinsulinaemia or sh‐YAP, the mRNA levels of hepatic SREBP‐1c and SREBP‐2 were apparently increased by the HFHS diet and reduced by sh‐YAP (Figure 4H). These results suggest that inhibition of YAP down‐regulates SREBP‐1c and SREBP‐2 and thereby inhibits the feed‐forward transcription of their own genes. In accordance with dynamically altered SREBP‐1c expression, the mRNA expression of its target genes, including acetyl‐CoA carboxylase 1 (ACC1), fatty acid synthase (FAS) and stearoyl CoA desaturase 1 (SCD1), which are enzymes involved in fatty acid and triglyceride synthesis, was obviously increased in HFHS‐fed mice and reduced apparently by administration of sh‐YAP (Figure 4H). Furthermore, due to the decreased SREBP‐2, the mRNA levels of two critical enzymes of cholesterol biosynthesis, namely, 30‐hydroxylmethyl glutaryl coenzyme A synthase (HMGCS) and 30‐hydroxylmethyl glutaryl coenzyme A reductase (HMGCR), were decreased in diabetic mice with sh‐YAP (Figure 4H).

These researches show that YAP knockdown decreases SREBPs expression and down‐regulates lipogenesis and cholesterol synthesis by suppressing auto‐loop regulation of SREBPs and decreasing their target genes expression.

### 3.4 YAP binds to SREBPs and functions as a transcriptional co‐activator of SREBPs

**Figure 5:**
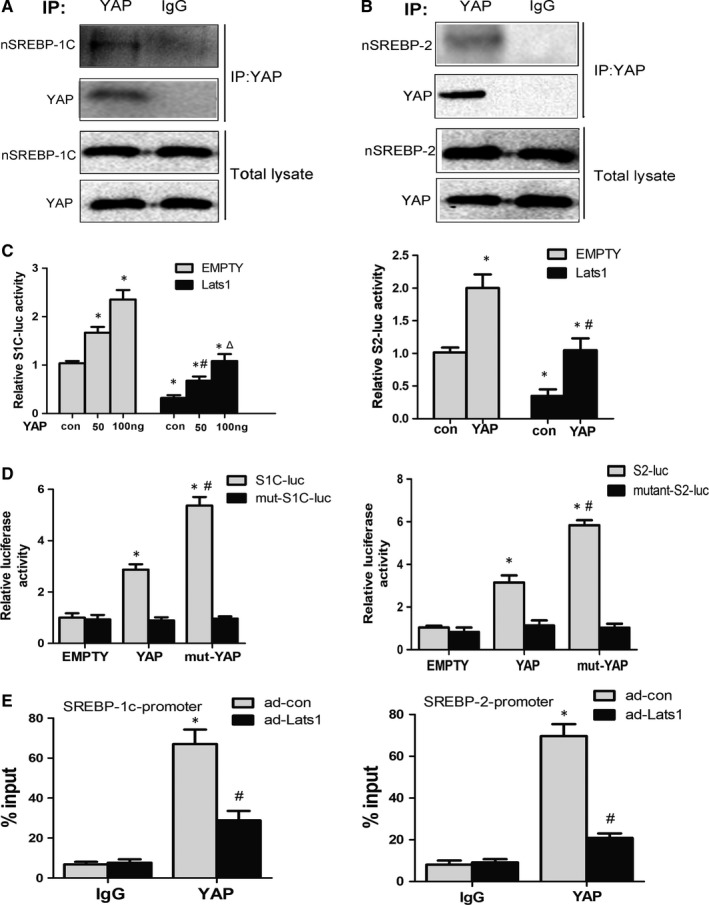
YAP associates with the nuclear forms of SREBP‐1 and SREBP‐2 and enhances their activities in hepatocytes. A and B, Primary mouse hepatocytes were prepared and immunoprecipitated with YAP antibody or control IgG. The interaction between SREBP‐1c, SREBP‐2 and YAP was examined. Immunoblots of input lysate controls (5% of inputs) are also shown. C, HepG2 cells were co‐transfected with SREBP‐1c‐luc vector and the indicated expression vectors. After 48 h, the luciferase activity was measured, demonstrating that YAP enhanced SREBP‐1c transcriptional activity. Overexpression of Lats1 inhibited SREBP‐1c activity (*P < 0.05 vs empty vector, ^#^ *P* < 0.05 vs empty vector plus 50 ng YAP, Δ*P* < 0.05 vs empty vector plus 100 ng YAP, mean ± SEM, n = 3). HepG2 cells were co‐transfected with SREBP‐2‐luc vector and the indicated expression vectors. After 48 h, the luciferase activity was measured, demonstrating that YAP enhanced SREBP‐2 transcriptional activity. Overexpression of Lats1 inhibited SREBP‐2 activity (*P < 0.05 vs empty vector, ^#^ *P* < 0.05 vs empty vector plus YAP, mean ± SEM, n = 3). D, YAP or mut‐YAP was co‐transfected with SREBP‐1c‐luc/SREBP‐2‐luc vector or mut‐ SREBP‐1c‐luc/SREBP‐2‐luc vector in HepG2 cells. After 48 h, the luciferase activity was measured (*P < 0.05 vs empty vector plus S1C‐luc, ^#^ *P* < 0.05 vs YAP plus S1C‐luc, mean ± SEM, n = 3). E, ChIP analysis of in vivo YAP binding to the SREBP‐1c and SREBP‐2 promoters. Primary hepatocytes were transfected with indicated adenovirus for 48 h. Protein‐bound chromatin was prepared and immunoprecipitated with IgG and YAP antibodies. Lats1 overexpression attenuated the binding. The relative occupancy on the promoters was compared with the input signal (*P < 0.05 vs ad‐con plus IgG, ^#^ *P* < 0.05 vs ad‐con plus YAP, mean ± SEM, n = 3)

Since the terminal effector of Hippo signalling pathway, YAP, always functions as a co‐activator of the transcription factor, we performed co‐immunoprecipitation assays to confirm that endogenous YAP associates with SREBP‐1c and SREBP‐2 in mouse liver hepatocytes (Figure 5A,B). Furthermore, IP showed the pYAP did not bind to SREBPs, demonstrating that only the dephosphorylated and activated YAP could associate with SREBPs (Supplementary Figure S2H). Then, we evaluated transcriptional activity of the SREBP1c‐luciferase reporter gene (S1C‐luc) driven by three canonical SREBP‐1cresponse elements (SREs). YAP stimulated SREBP‐1c transcriptional activity in a dose‐dependent manner, and this effect was reduced by overexpressing Lats1 (Figure 5C). Similarly, Lats1 could reverse the stimulation effect of YAP on SREBP‐2‐luc (S2‐luc) (Figure 5C). Moreover, mut‐YAP (S127A), not being dephoryphoslated, could also increase more activity of S1C‐luc and S2‐luc, compared with the wild‐type YAP (Figure 5D). As SREBPs could bind to the promoter region of their own via a feed‐forward mechanism, chromatin immunoprecipitation (ChIP) was conducted to confirm YAP present at the same region in primary mice hepatocytes. ChIP results showed that YAP was shown at the SREBP‐binding motifs in the promoter regions of SREBPs and Lats1 overexpression could inhibit the process (Figure 5E). Taken together, these data indicate that YAP binds to SREBP‐1c and SREBP‐2, and acts as a transcriptional co‐activator of these two transcriptional factors.

### 3.5 The YAP‐SREBPs complex mediates SREBP target genes expression in vitro

**Figure 6:**
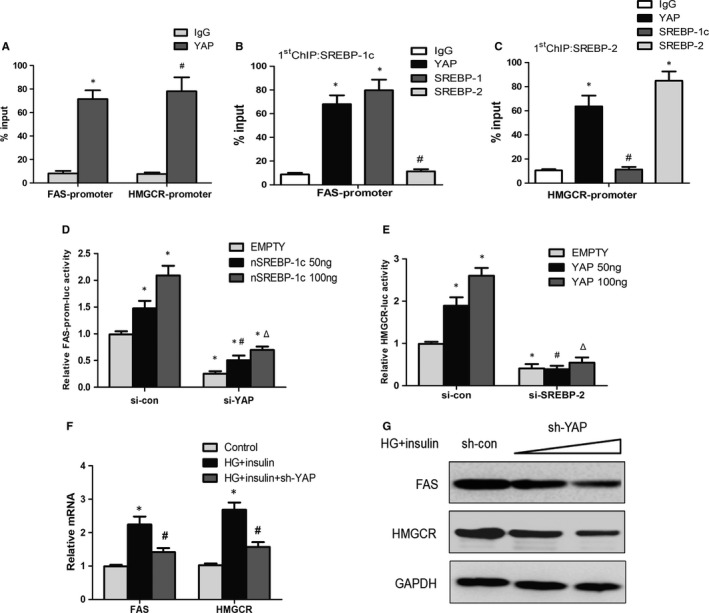
YAP is essential for SREBPs‐mediated genes expression in vitro. A, ChIP analysis of in vivo YAP and SREBP‐1c and SREBP‐2 binding to the FAS and HMGCR promoters. Protein‐bound chromatin was prepared from primary hepatocytes and immunoprecipitated with IgG and YAP antibodies. The relative occupancy on the promoters was compared with the input signal (*P < 0.05 vs IgG (FAS‐promoter), ^#^ *P* < 0.05 vs IgG (HMGCR‐promoter), mean ± SEM, n = 3). B and C, For sequential ChIP, the protein‐bound chromatin was first immunoprecipitated with the SREBP‐1c or SREBP‐2 antibody in primary hepatocytes. The second ChIP was then performed on the chromatin eluted from the first ChIP by immunoprecipitating it with the YAP, SREBP‐1c or SREBP‐2 antibody. Normal IgG was used as a negative control. The relative occupancy on the promoters was compared with the input signal (*P < 0.05 vs IgG, ^#^ *P* < 0.05 vs SREBP‐1c (B) or SREBP‐2 (C), mean ± SEM, n = 3). D, HepG2 cells were transfected with the FAS‐luc vector and the indicated vectors and siRNAs. After 48 h, luciferase activity was measured (*P < 0.05 vs empty vector plus si‐con, ^#^ *P* < 0.05 vs 50 ng nSREBP‐1c plus si‐con, Δ*P* < 0.05 vs 100 ng nSREBP‐1c plus si‐con, mean ± SEM, n = 3). E, HepG2 cells were transfected with the HMGCR‐luc vector and the indicated vectors and siRNAs. After 48 h, luciferase activity was measured (*P < 0.05 vs empty vector plus si‐con, ^#^ *P* < 0.05 vs 50 ng YAP plus si‐con, Δ*P* < 0.05 vs 100 ng YAP plus si‐con, mean ± SEM, n = 3). F, Primary hepatocytes transfected with the sh‐YAP were exposed to high glucose (HG) plus insulin. After 24 h, mRNA levels of FAS and HMGCR were detected by RT‐PCR (*P < 0.05 vs control group, ^#^ *P* < 0.05 vs HG + insulin group, mean ± SEM, n = 3). G, Protein levels of FAS and HMGCR exposed to HG plus insulin were inhibited by sh‐YAP treatment in primary hepatocytes.

To determine the role of YAP in SREBP target genes regulation, ChIP was performed with SREBP‐1c, SREBP‐2 and YAP antibodies in primary mice hepatocytes. The SREBP‐binding motifs present at the promoter of FAS and HMGCR, are essential for their transcriptional regulation in response to insulin.^2^ ChIP experiments revealed that YAP was present at the SREBP‐binding motifs at the promoter regions of both FAS and HMGCR (Figure 6A). To determine whether YAP was binding to SREBP‐1c or SREBP‐2 on the promoters of FAS or HMGCR, respectively, sequential ChIP experiments were performed. SREBP‐1c and SREBP‐2 antibodies were used to conduct the first ChIP, respectively. Then chromatin obtained from the first ChIP was applied to perform the second ChIP with YAP or SREBP antibodies. Results indicated that YAP and SREBPs were located at the same promoter regions of FAS and HMGCR, showing that YAP could bind to the SREBPs and form a complex at their promoters (Figure 6B,C). To further examine the functional role of the complex to regulate FAS and HMGCR, luciferase vectors of FAS (FAS‐luc) and HMGCR (HMGCR‐luc), containing one SREBP DNA‐binding site, were generated. Dual luciferase assay showed SREBP‐1c overexpression enhanced the FAS‐luc luciferase activity, and YAP knockdown partially attenuated this effect (Figure 6D). SREBP‐2 depletion could also abolish the enhancement of YAP on HMGCR‐luc activity (Figure 6E). RT‐PCR and Western blot showed YAP knockdown also apparently decreased the mRNA and protein levels of FAS and HMGCR (Figure 6F,G). These data demonstrate that YAP is an essential co‐activator of SREBP‐1c and SREBP‐2 and controls FAS and HMGCR genes expression in hepatocytes.

### 3.6 Hippo signalling suppresses SREBPs target genes expression

**Figure 7:**
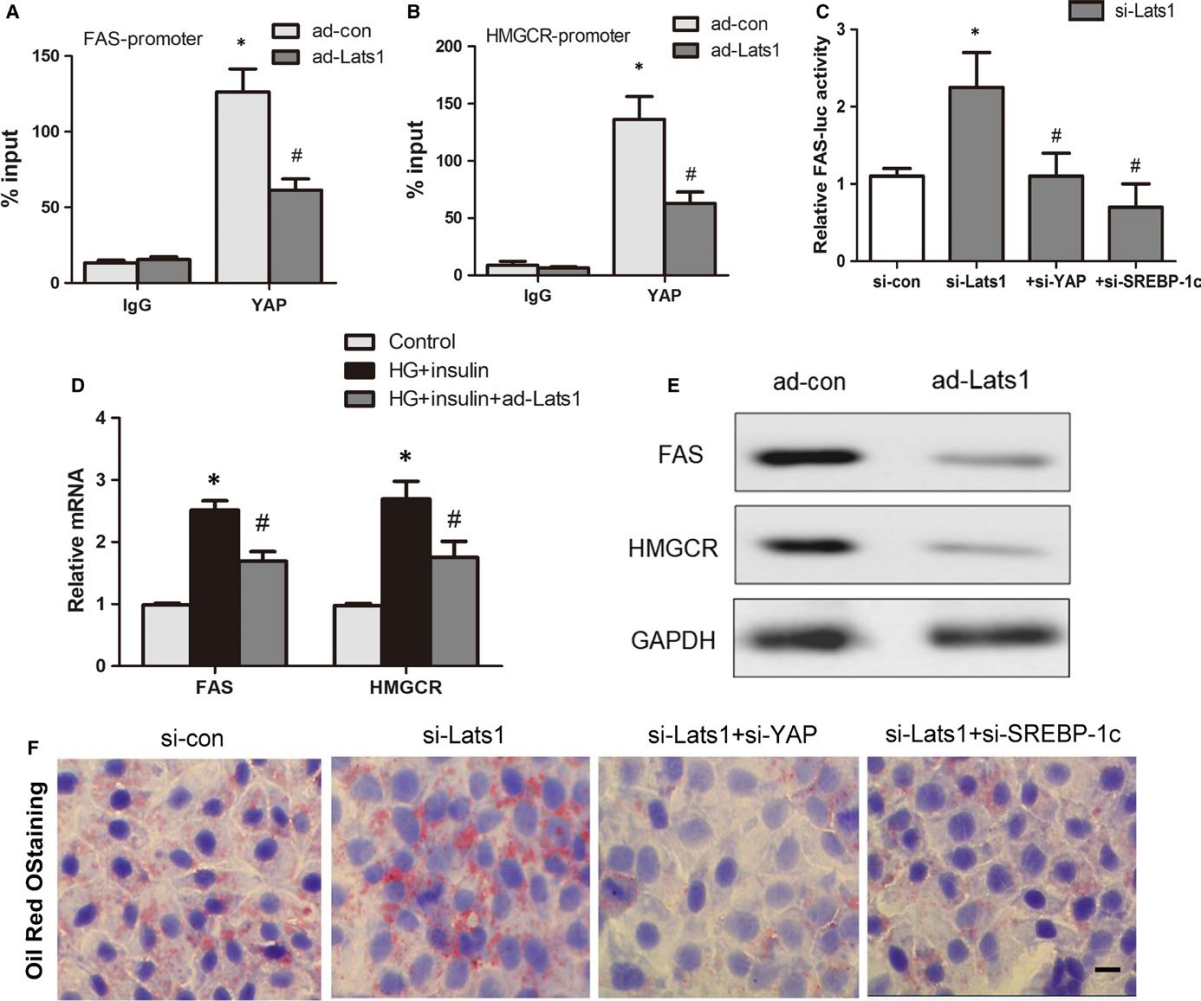
Hippo inactivation promotes SREBPs activity and hepatocyte lipogenesis in vitro. A and B, ChIP analysis of in vivo YAP binding to the FAS and HMGCR promoters. Primary hepatocytes were transfected with the indicated adenovirus for 48 h. Protein‐bound chromatin was prepared and immunoprecipitated with IgG and YAP antibodies. Lats1 overexpression attenuated the binding. The relative occupancy on the promoters was compared with the input signal (*P < 0.05 vs ad‐con plus IgG, ^#^ *P* < 0.05 vs ad‐con plus YAP, mean ± SEM, n = 3). C, HepG2 cells were transfected with the FAS‐luc vector and the indicated si‐RNAs. After 48 h, luciferase activity was measured (*P < 0.05 vs si‐con, ^#^ *P* < 0.05 vs si‐Lats1, mean ± SEM, n = 3). D, Overexpression of Lats1 reduced FAS and HMGCR mRNA and protein levels. Primary hepatocytes were exposed to high glucose (HG) plus insulin transfected with the ad‐Lats1. After 24 h, mRNA levels of FAS and HMGCR were detected by RT‐PCR (*P < 0.05 vs control group, ^#^ *P* < 0.05 vs HG + insulin group, mean ± SEM, n = 3). E, Protein levels of FAS and HMGCR exposed to HG plus insulin were inhibited by ad‐Lats treatment in primary hepatocytes. F, Knockdown of Lats1 increase lipid accumulation in HepG2 cells exposed to HG plus insulin, as reflected by oil red O staining, whereas knockdown of YAP or SREBP‐1c reverse this effect. Photographs were taken at magnifications of ×400, scale bars, 50 μm

We next explored the way Hippo signalling pathway regulates FAS and HMGCR expression. ChIP assays showed that Lats1 overexpression apparently decreased the quantity of YAP present at the SREBP‐binding sites in the promoter regions of FAS and HMGCR (Figure 7A,B). Lats1 knockdown in HepG2 cells enhanced the FAS–luc transcriptional activity, and this effect was completely abolished by down‐regulation of either YAP or SREBP‐1c at the same time (Figure 7C). Furthermore, as high glucose (30 mmol/L) and insulin increase the mRNA levels of both FAS and HMGCR through nuclear translocation of SREBPs, Lats1 overexpression reversed these effects (Figure 7D,E). FAS and HMGCR can effectively promote lipogenesis and cholesterol synthesis, leading to the formation of lipid droplets in HepG2 cells exposed to high glucose and insulin. And lipid accumulation was substantially increased by Lats1 knockdown, an effect that was completely reversed by simultaneous down‐regulation of YAP or SREBP‐1c in HepG2 cells in response to glucose and insulin treatment (Figure 7F). These results suggest that Hippo signalling activation impairs lipogenesis through suppression of YAP‐SREBP complexes.

## 4 DISCUSSION

Our results reveal that there is a close connection between the Hippo‐YAP signalling pathway and SREBPs in lipid metabolism of hepatocytes. We discovered that YAP is a novel coactivator of SREBP‐1c and SREBP‐2 and that the YAP‐SREBP complexes bind to their own and their target genes’ promoters –including FAS and HMGCR—a step that is essential for expression of key enzymes for lipogenesis and cholesterol synthesis in hepatocytes. Activation of the Hippo‐YAP signalling pathway by Lats1 overexpression or YAP knockdown in diet induced diabetic mice inhibits the YAP‐SREBP complexes and inactivates expression of SREBP target genes, thereby down‐regulating hepatic and plasma triglyceride and cholesterol levels. This down‐regulation of lipid levels alleviates hepatic steatosis and insulin resistance in diabetic mice.

Recent researches indicate that YAP activity is regulated by the SREBP/mevalonate pathway in many tumour cells, such as breast cancer cells. Increased mevalonic acid could promote YAP nuclear translocation and regulate tumour cells proliferation. Oncogenic mutant p53, acting as a positive transcriptional cofactor for SREBPs, leads to the increased mevalonic acid and promotes YAP activity in tumour cells.^17^ These results reveal a tight connection between YAP activity and cell lipid and cholesterol metabolism.

In our researches, we discover that YAP could also be activated by mevalonic acid in primary mice hepatocytes. And in mice fed with HFHS diet, YAP is activated and translocated from cytoplasm into nucleus of hepatocytes, which may be attributed to the increased plasm and liver cholesterol. Although the mevalonic acid regulates YAP phosphorylation and activity independently of Lats1/2 kinases in tumour cells, the directly upstream regulator of the pathway effector YAP is also dephosphorylated and inactivated in HFHS fed mice, contributing to the YAP nuclear translocation and activation. The mechanism of dephosphorylation of both Lats1/2 and Mst1/2 still need exploring. In line with this, statins, inhibiting the enzyme HMG‐CoA reductase to lower cellular cholesterol levels, may also regulate YAP nuclear translocation in normal hepatocytes. These results manifest that Hippo‐YAP signalling may have important implications in metabolic diseases.

YAP, which binds to many transcription factors including the Runx family and TEAD, is negatively regulated by the Hippo signalling pathway and has redundant roles in several biological events, including tumorigenesis.^33^, ^34^ However, there has been no research into the role of Hippo signalling in cell metabolism. SREBP‐1c and SREBP‐2 are important transcription factors in lipid and cholesterol metabolism of hepatocytes, which plays a critical role in diabetic mice. In diabetic mice, Hippo signalling is inhibited and YAP is dephosphorylated and translocates into the nucleus of hepatocytes, functioning as a co‐activator of SREBP‐1c and SREBP‐2, which contributes to lipogenesis and cholesterol synthesis. As the relationship of Hippo pathway and many other nutrients metabolism still remains unclear, it would be interesting to explore the role of Hippo‐YAP signalling in other cell metabolic processes, such as proteometabolism and glycometabolism. Whether YAP could be a regulator for these processes needs further research.

In a recent research, Lats2 was shown to inhibit SREBPs and suppresses hepatic cholesterol accumulation through a Lats2‐p53 axis.^35^ Mice harbouring liver‐specific Lats2 conditional knockout displayed SREBP activation, leading to spontaneous fatty liver disease.^35^ Furthermore, only Lats2, not Lats1, could inhibit SREBPs and suppress hepatic cholesterol accumulation. This phenomenon suggests that the directly upstream regulator of YAP can suppress SREBPs independently of Hippo‐YAP signalling. Here, we showed that both MVA stimulation and Lats1 dephosphorylation in HFHS diet fed mice could activate YAP and promote SREBPs transcriptional activity in mice. So, SREBPs may be regulated by the components of the Hippo signalling through different mechanism, which may be stimulus‐specific or coordinately regulated by the triglyceride and cholesterol metabolism of hepatocytes.

Cancer cells share many common characteristic alterations in metabolism. Cellular proliferation is the common feature of all cancer cells, requiring fatty acids for synthesis of membranes and signalling molecules.^36^ Enhanced denovo lipogenesis is a key feature of cancer cells. Key enzymes related to fatty acid synthesis, such as FAS, ACC and SCD, are highly expressed in many cancer cells. The master transcriptional regulator is SREBP‐1c, and suppressing SREBP‐1c in cancer cells prevents fatty acid synthesis, leading to inhibition of cancer cell proliferation.^37^, ^38^ In addition, increasing lines of evidence suggest that the hyperactivation of YAP, due to inactivation of Hippo signalling, promotes cell proliferation and acts as a tumour promoter in many organs.^7^ Thus, it is tempting to speculate that YAP‐SREBPs complex may regulate tumorigenesis through controlling cell lipogenesis genes.

LXRα regulates fatty acid and triglyceride synthesis, as it induces SREBP‐1c activation and expression via an LXR response element in its promoter.^41^, ^42^ mTORC1 is also an critical controller of SREBP‐1c since it can activate SREBP‐1c transcription in response to insulin stimulation.^43^ Here, we show that YAP functions as a coactivator of SREBPs and YAP knockdown decreases their activities. These multiple transcriptional controllers of SREBPs may control their expression in a coordinated and stimulus‐specific manner.

It is known that SREBPs are associated with the SREBP cleavage activating protein (SCAP) and ER retention protein called Insig.^44^ To be activated, the SREBP‐SCAP complex should dissociate from Insig, associate with COPII‐coated vesicles and then migrate to the Golgi apparatus for proteolytic activation.^45^ We here showed that diabetic mice with ad‐Lats1 or sh‐YAP share some features with SCAP knockout mice or transgenic mice overexpressing Insig‐1, decreased nSREBPs.^46^, ^47^ In addition to the transcriptional regulation, it would be of interest to determine whether Hippo signalling regulates SREBPs proteolytic processing in hepatocytes.

In conclusion, our research reveals a novel relationship between the Hippo pathway and SREBPs through YAP, and describes the mechanism of this regulation in the pathogenesis of diabetic mice. We speculate that the connection between YAP and SREBPs is a point of convergence that allows Hippo pathway to precisely control many biological processes in metabolic disease.

## CONFLICT OF INTEREST

The authors declare that there is no conflict of interest that could be perceived as prejudicing the impartiality of the research reported.

## AUTHORS' CONTRIBUTIONS

Wenqian Xiong and Zhiping Shu designed and performed the research. Wenqian Xiong analysed the data and wrote the paper. Xiaohua Zhu and Yuan Gao contributed the animal study. Guopeng Zhang, Yu Zhou, Jing Cao and Dongyi Wan reviewed the manuscript. All authors read and approved the final manuscript.

## References

[1] Semenkovich CF . Insulin resistance and atherosclerosis. J Clin Invest. 2006;116:1813‐1822. 16823479 10.1172/JCI29024 PMC1483180

[2] Horton JD , Goldstein JL , Brown MS . SREBPs: activators of the complete program of cholesterol and fatty acid synthesis in the liver. J Clin Invest. 2002;109:1125‐1131. 11994399 10.1172/JCI15593 PMC150968

[3] Radhakrishnan A , Goldstein JL , McDonald JG , Brown MS . Switch‐like control of SREBP‐2 transport triggered by small changes in ER cholesterol: a delicate balance. Cell Metab. 2008;8:512‐521. 19041766 10.1016/j.cmet.2008.10.008 PMC2652870

[4] Stoeckman AK , Towle HC . The role of SREBP‐1c in nutritional regulation of lipogenic enzyme gene expression. J Biol Chem. 2002;277:27029‐27035. 12016216 10.1074/jbc.M202638200

[5] Sakakura Y , Shimano H , Sone H , et al. Sterol regulatory element‐binding proteins induce an entire pathway of cholesterol synthesis. Biochem Biophys Res Commun. 2001;286:176‐183. 11485325 10.1006/bbrc.2001.5375

[6] Eberlé D , Hegarty B , Bossard P , Ferré P , Foufelle F . SREBP transcription factors: master regulators of lipid homeostasis. Biochimie. 2004;86:839‐848. 15589694 10.1016/j.biochi.2004.09.018

[7] Pan D . The hippo signaling pathway in development and cancer. Dev Cell. 2010;19:491‐505. 20951342 10.1016/j.devcel.2010.09.011 PMC3124840

[8] Edgar BA . From cell structure to transcription: Hippo forges a new path. Cell. 2006;124:267‐273. 16439203 10.1016/j.cell.2006.01.005

[9] Harvey K , Tapon N . The Salvador‐Warts‐Hippo pathway ‐ an emerging tumour‐suppressor network. Nat Rev Cancer. 2007;7:182‐191. 17318211 10.1038/nrc2070

[10] Camargo FD , Gokhale S , Johnnidis JB , et al. YAP1 increases organ size and expands undifferentiated progenitor cells. Curr Biol. 2007;17:2054‐2060. 17980593 10.1016/j.cub.2007.10.039

[11] Zhao B , Wei X , Li W , et al. Inactivation of YAP oncoprotein by the Hippo pathway is involved in cell contact inhibition and tissue growth control. Genes Dev. 2007;21:2747‐2761. 17974916 10.1101/gad.1602907 PMC2045129

[12] Zhou D , Conrad C , Xia F , et al. Mst1 and Mst2 maintain hepatocyte quiescence and suppress hepatocellular carcinoma development through inactivation of the Yap1 oncogene. Cancer Cell. 2009;16:425‐438. 19878874 10.1016/j.ccr.2009.09.026 PMC3023165

[13] Zhang WQ , Dai YY , Hsu PC , et al. Targeting YAP in malignant pleural mesothelioma. J Cell Mol Med. 2017;21:2663‐2676. 28470935 10.1111/jcmm.13182 PMC5661117

[14] Hong JH , Hwang ES , McManus MT , et al. TAZ, a transcriptional modulator of mesenchymal stem cell differentiation. Science. 2005;309:1074‐1078. 16099986 10.1126/science.1110955

[15] Nishioka N , Inoue K‐I , Adachi K , et al. The Hippo signaling pathway components Lats and Yap pattern Tead4 activity to distinguish mouse trophectoderm from inner cell mass. Dev Cell. 2009;16:398‐410. 19289085 10.1016/j.devcel.2009.02.003

[16] Dong J , Feldmann G , Huang J , et al. Elucidation of a universal size‐control mechanism in Drosophila and mammals. Cell. 2007;130:1120. 17889654 10.1016/j.cell.2007.07.019 PMC2666353

[17] Sorrentino G , Ruggeri N , Specchia V , et al. Metabolic control of YAP and TAZ by the mevalonate pathway. Nat Cell Biol. 2014;16:357‐366. 24658687 10.1038/ncb2936

[18] Huang W , Dedousis N , O'Doherty RM . Hepatic steatosis and plasma dyslipidemia induced by a high‐sucrose diet are corrected by an acute leptin infusion. J Appl Physiol. 2007;102:2260‐2265. 17363621 10.1152/japplphysiol.01449.2006

[19] Li Yu , Xu S , Mihaylova MM , et al. AMPK phosphorylates and inhibits SREBP activity to attenuate hepatic steatosis and atherosclerosis in diet‐induced insulin resistant mice. Cell Metab. 2011;13:376‐388. 21459323 10.1016/j.cmet.2011.03.009 PMC3086578

[20] Radaeva S , Jaruga B , Hong F , et al. Interferon‐α activates multiple STAT signals and down‐regulates c‐Met in primary human hepatocytes. Gastroenterology. 2002;122:1020‐1034. 11910354 10.1053/gast.2002.32388

[21] Matsui Y , Nakano N , Shao D , et al. Lats2 is a negative regulator of myocyte size in the heart. Circ Res. 2011;103:1309‐1318. 10.1161/CIRCRESAHA.108.180042 PMC2775813 18927464

[22] Shao D , Zhai P , Re D , et al. A functional interaction between hippo‐YAP signaling and Foxo1 mediates the oxidative stress response. Nat Commun. 2014;5:3315. 24525530 10.1038/ncomms4315 PMC3962829

[23] Sato R , Inoue J , Kawabe Y , Kodama T , Takano T , Maeda M . Sterol‐dependent transcriptional regulation of sterol regulatory element‐binding protein‐2. J Biol Chem. 1996;271:26461‐26464. 8900111 10.1074/jbc.271.43.26461

[24] Dif N , Euthine V , Gonnet E , Laville M , Vidal H , Lefai E . Insulin activates human sterol‐regulatory‐element‐binding protein‐1c (SREBP‐1c) promoter through SRE motifs. Biochem J. 2006;400:179‐188. 16831124 10.1042/BJ20060499 PMC1635455

[25] Howe V , Sharpe LJ , Prabhu AV , et al. New insights into cellular cholesterol acquisition: Promoter analysis of human HMGCR and SQLE, two key control enzymes in cholesterol synthesis. Biochem Biophys Acta. 2017;1862:647-657. 10.1016/j.bbalip.2017.03.009 28342963

[26] Joseph SB , Laffitte BA , Patel PH , et al. Direct and indirect mechanisms for regulation of fatty acid synthase gene expression by liver X receptors. J Biol Chem. 2002;277:11019. 11790787 10.1074/jbc.M111041200

[27] Schreyer SA , Chua S , Leboeuf RC . Obesity and diabetes in TNF‐α receptor‐ deficient mice. J Clin Invest. 1998;102:402‐411. 9664082 10.1172/JCI2849 PMC508899

[28] Surwit Rs , Seldin Mf , Kuhn Cm , Cochrane C , Feinglos Mn . Control of expression of insulin resistance and hyperglycemia by different genetic factors in diabetic C57BL/6J mice. Diabetes. 1991;40:82. 2015977 10.2337/diab.40.1.82

[29] Schreyer SA , Wilson DL , Leboeuf RC . C57BL/6 mice fed high fat diets as models for diabetes‐accelerated atherosclerosis. Atherosclerosis. 1998;136:17‐24. 9544727 10.1016/s0021-9150(97)00165-2

[30] Surwit Rs , Kuhn Cm , Cochrane C , McCubbin Ja , Feinglos Mn . Diet‐induced type II diabetes in C57BL/6J mice. Diabetes. 1988;37:1163‐1167. 3044882 10.2337/diab.37.9.1163

[31] Meng Z , Moroishi T , Guan KL . Mechanisms of Hippo pathway regulation. Genes Dev. 2016;30:3616‐17. 10.1101/gad.274027.115 PMC4701972 26728553

[32] Van Rooyen DM , Farrell GC . SREBP‐2: a link between insulin resistance, hepatic cholesterol, and inflammation in NASH. J Gastroenterol Hepatol. 2011;26:789‐792. 21488942 10.1111/j.1440-1746.2011.06704.x

[33] Zhang H , Liu C‐Y , Zha Z‐Y , et al. TEAD transcription factors mediate the function of TAZ in cell growth and epithelial‐mesenchymal transition. J Biol Chem. 2009;284:13355‐13362. 19324877 10.1074/jbc.M900843200 PMC2679435

[34] Zhao B , Lei QY , Guan KL . The Hippo–YAP pathway: new connections between regulation of organ size and cancer. Curr Opin Cell Biol. 2008;20:638. 18955139 10.1016/j.ceb.2008.10.001 PMC3296452

[35] Aylon Y , Gershoni A , Rotkopf R , et al. The LATS2 tumor suppressor inhibits SREBP and suppresses hepatic cholesterol accumulation. Genes Dev. 2016;30:786. 27013235 10.1101/gad.274167.115 PMC4826395

[36] Currie E , Schulze A , Zechner R , Walther T , Farese R . Cellular fatty acid metabolism and cancer. Cell Metab. 2013;18:153‐161. 23791484 10.1016/j.cmet.2013.05.017 PMC3742569

[37] Guo D , Prins RM , Dang J , et al. EGFR signaling through an Akt‐SREBP‐1‐dependent, rapamycin‐resistant pathway sensitizes glioblastomas to antilipogenic therapy. Science Signaling. 2009;2:ra82. 20009104 10.1126/scisignal.2000446 PMC2978002

[38] Ettinger SL , Sobel R , Whitmore TG , et al. Dysregulation of sterol response element‐binding proteins and downstream effectors in prostate cancer during progression to androgen independence. Can Res. 2004;64:2212‐2221. 10.1158/0008-5472.can-2148-2 15026365

[39] Williams Kj , Argus Jp , Zhu Y , et al. An essential requirement for the SCAP/SREBP signaling axis to protect cancer cells from lipotoxicity. Can Res. 2013;73:2850. 10.1158/0008-5472.CAN-13-0382-T PMC3919498 23440422

[40] Griffiths B , Lewis CA , Bensaad K , et al. Sterol regulatory element binding protein‐dependent regulation of lipid synthesis supports cell survival and tumor growth. Cancer Metab. 2013;1:3. 24280005 10.1186/2049-3002-1-3 PMC3835903

[41] Chen G , Liang G , Ou J , Goldstein JL , Brown MS . Central role for liver X receptor in insulin‐mediated activation of Srebp‐1c transcription and stimulation of fatty acid synthesis in liver. Proc Natl Acad Sci U S A. 2004;101(31):11245–11250 15266058 10.1073/pnas.0404297101 PMC509189

[42] Repa JJ , Liang G , Ou J , et al. Regulation of mouse sterol regulatory element‐binding protein‐1c gene (SREBP‐1c) by oxysterol receptors, LXRalpha and LXRbeta. Genes Dev. 2000;14:2819. 11090130 10.1101/gad.844900 PMC317055

[43] Porstmann T , Santos CR , Griffiths B , et al. SREBP activity is regulated by mTORC1 and contributes to Akt‐dependent cell growth. Cell Metab. 2008;8:224‐236. 18762023 10.1016/j.cmet.2008.07.007 PMC2593919

[44] Goldstein JL , Rawson RB , Brown MS . Mutant mammalian cells as tools to delineate the sterol regulatory element‐binding protein pathway for feedback regulation of lipid synthesis. Arch Biochem Biophys. 2002;397:139. 11795864 10.1006/abbi.2001.2615

[45] Sun L‐p , Seemann J , Goldstein Jl , Brown Ms . Sterol‐regulated transport of SREBPs from endoplasmic reticulum to Golgi: Insig renders sorting signal in Scap inaccessible to COPII proteins. Proc Natl Acad Sci U S A. 2007;104(16):6519–6526 17428919 10.1073/pnas.0700907104 PMC1851663

[46] Matsuda M , Korn BS , Hammer RE , et al. SREBP cleavage‐activating protein (SCAP) is required for increased lipid synthesis in liver induced by cholesterol deprivation and insulin elevation. Genes Dev. 2001;15:1206. 11358865 10.1101/gad.891301 PMC313801

[47] Engelking LJ , Kuriyama H , Hammer RE , et al. Overexpression of Insig‐1 in the livers of transgenic mice inhibits SREBP processing and reduces insulin‐stimulated lipogenesis. J Clin Invest. 2004;113:1168‐1175. 15085196 10.1172/JCI20978 PMC385408

