# Supplementary figures and images for: A functional interaction between Hippo‐YAP signalling and SREBPs mediates hepatic steatosis in diabetic mice
Source: J Cell Mol Med. 2019 Mar 1;23(5):3616–28. doi: 10.1111/jcmm.14262 (PMC6484311; doi:10.1111/jcmm.14262)

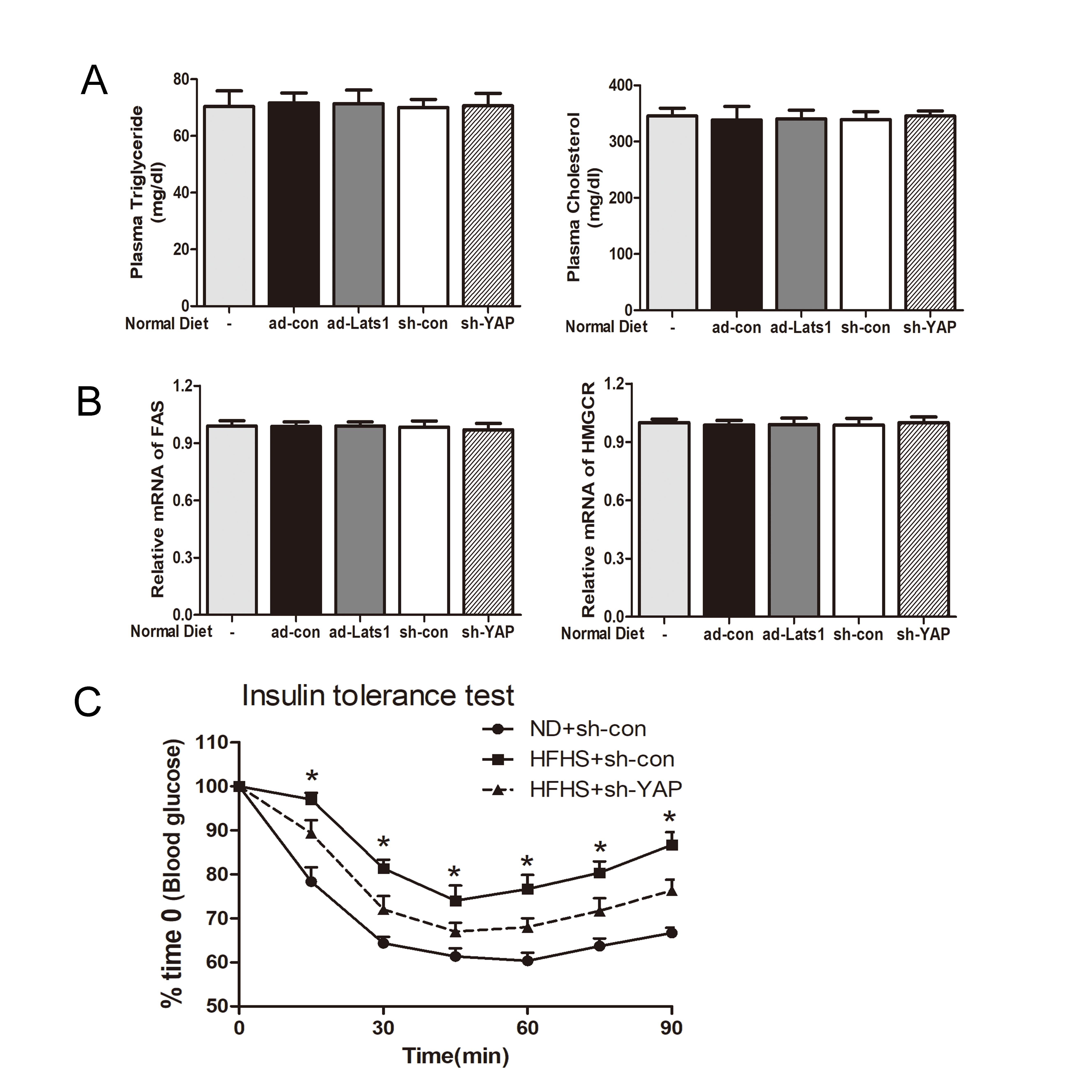

Supplement: Supplementary file 1 [file JCMM-23-3616-s001.tif]

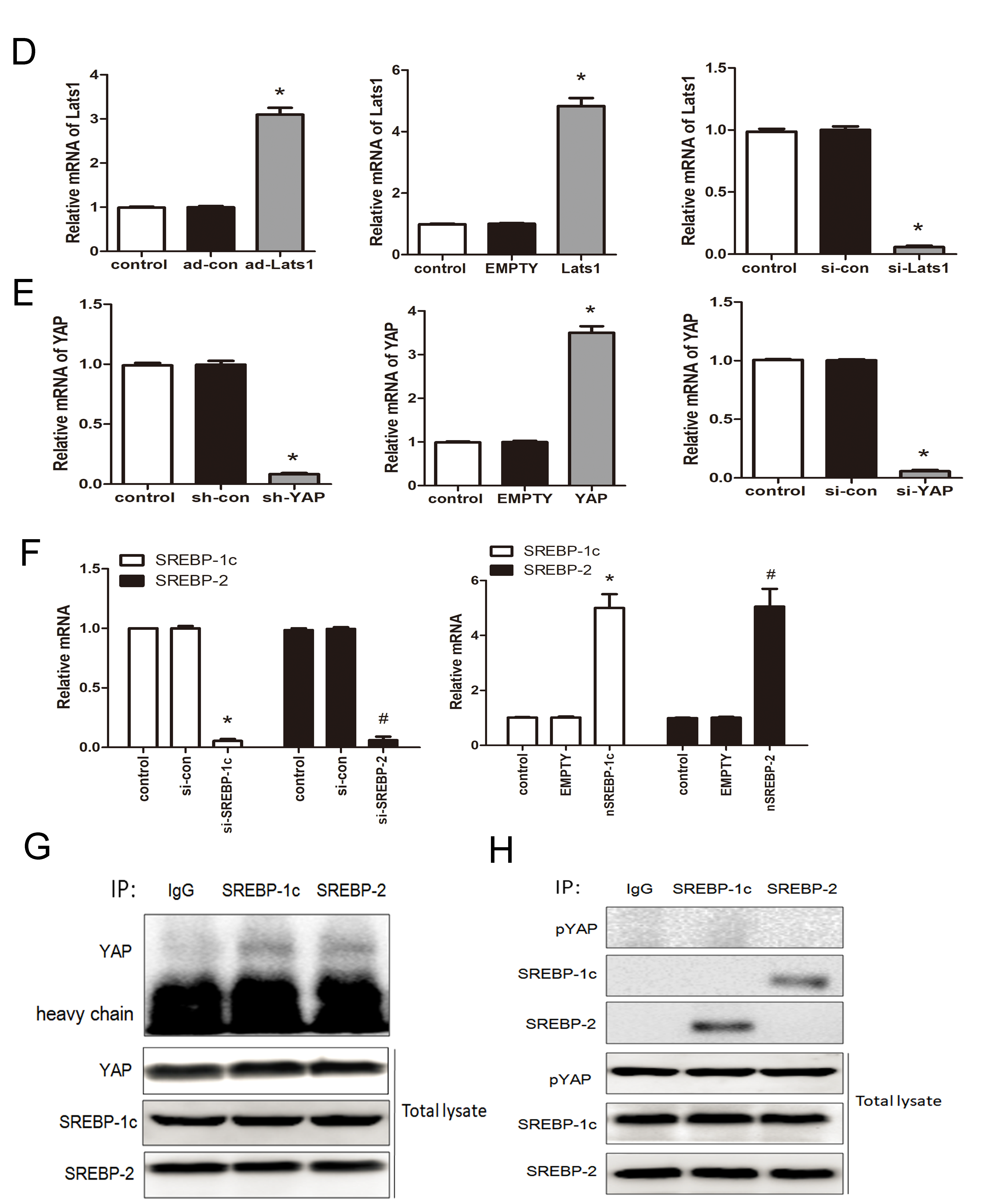

Supplement: Supplementary file 2 [file JCMM-23-3616-s002.tif]
